# Inhibition of LDHA suppresses tumor progression in prostate cancer

**DOI:** 10.1007/s13277-015-3540-x

**Published:** 2015-05-16

**Authors:** Zhi-Yong Xian, Jiu-Min Liu, Qing-Ke Chen, Han-Zhong Chen, Chu-Jin Ye, Jian Xue, Huan-Qing Yang, Jing-Lei Li, Xue-Feng Liu, Su-Juan Kuang

**Affiliations:** 1 Department of Urology, Guangdong General Hospital, 106 Zhongshan Second Road, Yuexiu District, Guangdong, China; 2 Department of Urology, Sihui City People’s Hospital, Guangdong, China; 3 Department of Radiology, Guangdong General Hospital, Guangdong, China; 4 Department of Pathology, Guangdong General Hospital, Guangdong, China; 5 Department of Medical Experimental Center, Guangdong General Hospital, Guangdong, China

**Keywords:** LDHA, Warburg effect, Prostate cancer, Progression

## Abstract

A key hallmark of cancer cells is their altered metabolism, known as Warburg effect. Lactate dehydrogenase A (LDHA) executes the final step of aerobic glycolysis and has been reported to be involved in the tumor progression. However, the function of LDHA in prostate cancer has not been studied. In current study, we observed overexpression of LDHA in the clinical prostate cancer samples compared with benign prostate hyperplasia tissues as demonstrated by immunohistochemistry and real-time qPCR. Attenuated expression of LDHA by siRNA or inhibition of LDHA activities by FX11 inhibited cell proliferation, migration, invasion, and promoted cell apoptosis of PC-3 and DU145 cells. Mechanistically, decreased Warburg effect as demonstrated by reduced glucose consumption and lactate secretion and reduced expression of MMP-9, PLAU, and cathepsin B were found after LDHA knockdown or FX11 treatment in PC-3 and DU145 cells. Taken together, our study revealed the oncogenic role of LDHA in prostate cancer and suggested that LDHA might be a potential therapeutic target.

## Introduction

Although great advances in medical management and screening, prostate cancer (PC) remains a leading cause of morbidity and mortality in men [1]. About 15 % of prostates contain islands of cancer at the age of 50 and nearly 100 % by 80 [2]. Nowadays, the chief treatment for PC is androgen deprivation therapy; however, patients on this regime eventually relapse into hormone-refractory prostate cancer. An enhanced tumor microenvironment that favors tumor progression is formed during the critical switch of PC to androgen independence and distant metastasis [3]. Therefore, it is urgent to identify key signaling events driving the tumor microenvironment to find novel therapeutic targets for PC.

Cancer cells take up glucose and transform it to lactate even under aerobic conditions, known as the Warburg effect. Sufficient amounts of nucleotides, proteins, and lipids derived from this type of glucose metabolism are required for cancer cell rapid growth and division [4]. Critical to this highly glycolytic phenotype is lactate dehydrogenase A (LDHA), which catalyses the last step of anaerobic glycolysis. Abnormally expression of LDHA has been observed in many human cancers, such as pancreatic cancer [5], hepatocellular carcinoma [6], and breast cancer [7]. Inhibition of LDHA reduced cell malignant transformation and remarkably delayed tumor formation, indicating that the underlying role of LDHA in tumor initiation or maintenance [8]. Several mechanisms by which LDHA suppression induces inhibition of tumor progression are revealed. In lymphoma, reduction of LDHA induces oxidative stress and alters cellular energy metabolism, which ultimately contributes to cell death [9]. In breast cancer, LDHA knockdown suppresses tumorigenicity through induction of oxidative stress mediated mitochondrial pathway apoptosis [10]. In PC, overexpressed lactate dehydrogenase 5 isoenzyme has been reported and confers prostate cancer with resistance of to radiotherapy [11]. However, little is known the expression pattern of LDHA and its underlying roles in PC.

In the present study, we firstly detected the expression of LDHA in PC and BPH tissues. Next, cellular functions of LDHA were analyzed in PC-3 and DU145 cells when LDHA expression was suppressed or LDHA activity was inhibited. Moreover, LDHA-dependent mechanisms involved in the progression of PC were also analyzed.

## Materials and methods

### Preparation of reagents and cell culture

FX11 (Merck Millipore, Germany) was dissolved in dimethylsulfoxide (DMSO) and further diluted to preferable concentrations in culture medium before use. Human prostate cancer cell lines 22Rv1, DU145, PC-3, and LNCaP were all purchased from Cell Bank of the Chinese Academy of Sciences. All cells were cultured in DMEM specific medium supplemented with 10 % (*v*/*v*) fetal calf serum at 37 °C in a humidified incubator under 5 % CO_2_ condition.

### Clinical tissue samples and immunohistochemistry

Twenty freshly frozen PC tissues and 12 benign prostate hyperplasia (BPH) tissues were recruited from Department of Urology, Guangdong General Hospital, China. All tissue samples were obtained with informed consent and approved by the ethics committee of Guangzhou Municipality. A tissue microarray containing 64 cases of PC tissues and 11 cases of BPH tissues were purchased from Xi-an Alenabio Inc. (China). After deparaffinizing, rehydrating, antigen retrieval, and neutralization of endogenous peroxidase, tissue sections were blocked with blocking serum, followed by incubation with primary antibody (LDHA, Proteintech) overnight at 4 °C. After washing in phosphate-buffered saline (PBS) for three times, the sections were incubated with HRP-labeled anti-rabbit secondary antibody. The reaction products were visualized with 3,3′-diaminobenzidine tetrahydrochloride (DAB) and counterstained by hematoxylin. Scoring was conducted on a scale of 0–3: 0–10 % scored 0; 10–35 % scored 1; 36–70 % scored 2; and more than 70 % scored 3. Scored at 0 and 1 was defined as low expression group, while 2 and 3 was defined as high expression group. The scoring by the pathologists was done in a blinded manner.

### siRNA transfection

PC-3 cells were transfected specific siRNAs targeting LDHA as well as a negative control (GenePharma, Shanghai, China). Transfection was accomplished by seeding 2 × 10^5^ cells into a six-well plate, and after 24 h, the medium was aspirated and incubated with transfection complex according to the manufacturer’s protocol. The interference efficiency was detected by Western blotting, and cell populations with lowest LDHA expression were used for subsequent experiments.

### Real-time quantitative PCR

Total RNA was isolated from cells or frozen tissue samples using TRIzol reagent (Invitrogen) according to the manufacturer’s instructions. Reverse transcription was performed using the PrimeScript RT-PCR system (Takara, Japan). Then 1 μg cDNA was quantified by real-time PCR with SYBR Green Master Mix (Takara, Japan). Specific primer sequences used were as follows: MMP9: forward 5′-GGGACGCAGACATCGTCATC-3′, reverse 5′-TCGTCATCGTCGAAATGGGC-3′; PLAU: forward 5′- GCTTGTCCAAGAGTGCATGGT -3′, reverse 5′-CAGGGCTGGTTCTCGATGG-3′; cathepsin B: forward 5′-AGAGTTATGTTTACCGAGGACCT-3′, reverse 5′-GATGCAGATCCGGTCAGAGA-3′. The expression level of gene analyzed in this study was standardized using the expression level of β-actin to obtain a relative level of gene expression.

### Western blotting analysis

Whole cell extracts were prepared using RIPA buffer supplemented with protease inhibitor (Beyotime, China). Protein lysates were separated by SDS-PAGE and target proteins were probed by Western blotting with antibodies (LDHA and β-actin, Proteintech). Then, the membranes were consecutively incubated with HRP-conjugated secondary antibodies (Abmart, China). The immunoreactive proteins were visualized by ECL Plus kit (Millipore Corporation, Billerica).

### Cell migration and invasion assays

Migration and invasion assay was performed with 8.0-μm pore inserts (Millipore, USA) in 24-well plate. For migration assay, 20,000 cells were seeded into the upper compartment of the transwell inserts. The invasion assay was performed with matrigel-coated filters (BD Bioscience, USA). Cells were allowed to incubate for 24 and 48 h, respectively. Migrated and invaded were fixed and stained by 0.1 % (*w*/*v*) crystal violet. Each experiment was performed in triplicate.

### Cell viability assay

Cell viability was evaluated by Cell Counting Kit-8 (CCK-8, Dojindo, Japan) following the manufacturer’s protocols. Briefly, 3000 cells were resuspended and seeded into a 96-well plate supplemented in the presence of 10 % FBS and cultured overnight. The next day, the LDHA knockdown cells or FX11-treated cells was incubated with CCK8 for 1 h and the absorbance was measured at 450 nm using a multifunctional microplate reader (Tecan). This experiment was done in quadruplicate cells.

### Apoptosis assay

Cells under apoptotic condition were analyzed by Annexin V/PI staining. Briefly, cells with small interfering (RNA) siRNA or FX11 treatment were seeded in six-well plates at 3 × 10^5^ cells per well in the absence of FBS for 48 h. Then, cells were harvested and labeled with FITC-conjugated Annexin V and propidium iodide (BD Pharmingen) following the manufacturer’s instructions. The apoptotic cells were measured by flow cytometry. The caspase-3/7 activity assay was performed at 48 h after serum deprivation according to manufacturer’s instructions (Promega)

### Measurements of lactate production and glucose consumption

Cells were cultured in fresh phenol red-free media and the culture media were collected in the first 24 h after siRNA or FX11 treatment. The glucose and glutamine concentration in the culture media are 5 and 2 mM, respectively. The lactate and glucose levels were measured by using lactate assay kits (Biovision) or glucose assay kits (Life Technologies), respectively.

### Statistical analysis

Data were presented as the means ± standard error of the mean (SEM). Statistical analyses and graphical representations were performed with SPSS 16.0 (SPSS Inc., Chicago, USA) and GraphPad Prism 5 (San Diego, CA) software. Cell viability assay was analyzed by one-way ANOVA. And, the Student’s *t* test was used for comparison between groups in other data. Values of *P* < 0.05 were considered statistically significant.

## Results

### Altered expression of LDHA were observed in PC

**Fig. 1:**
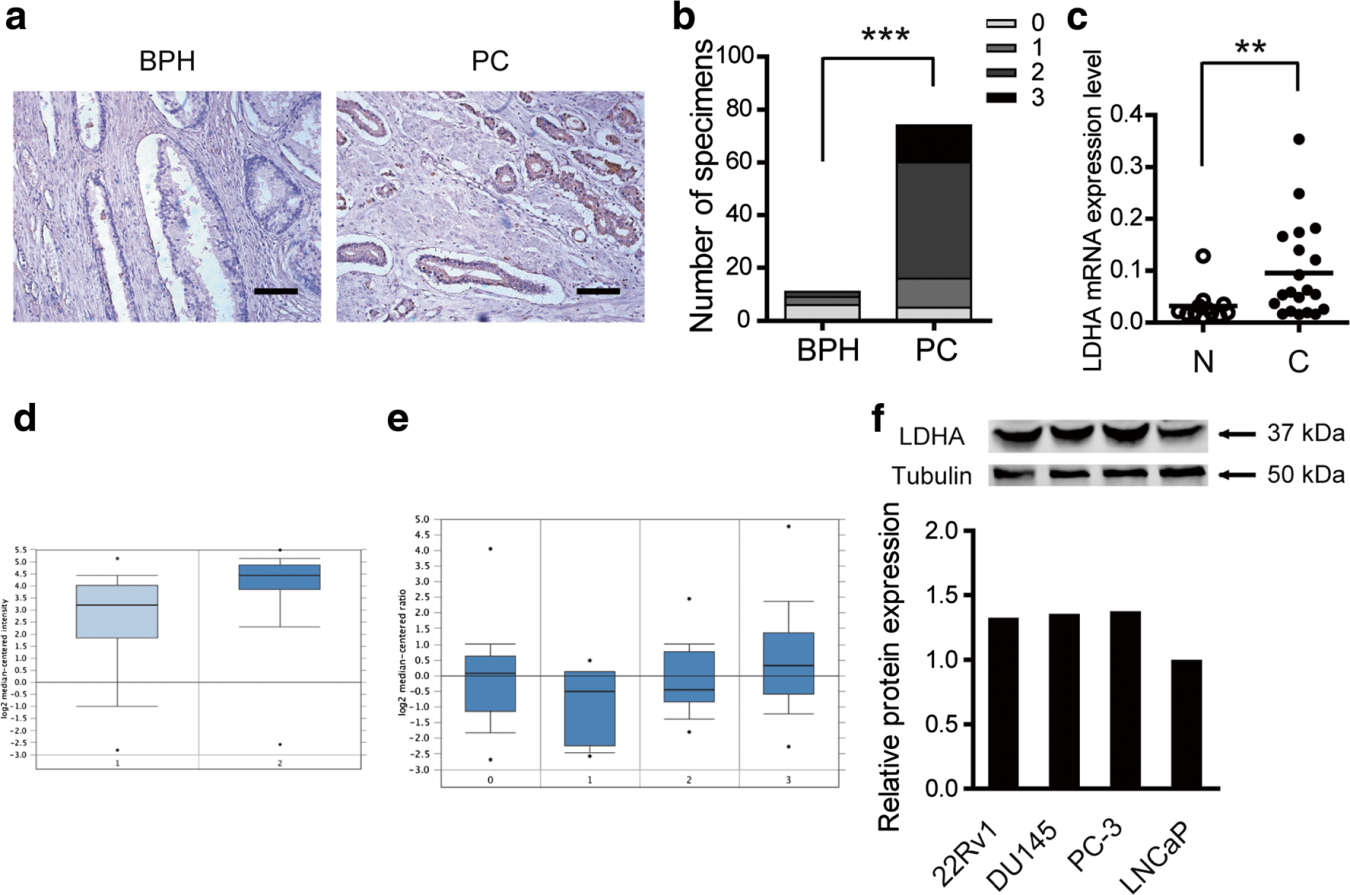
Altered expression of LDHA was observed in PC. **a** Representative images of the LDHA expression in benign prostate hyperplasia (BPH) and pancreatic cancer (PC); *scale bar*: 50 μm. **b** Increased LDHA protein expression in 11 BPH tissues and 74 PC tissues were detected by immunohistochemistry. **c** Increased LDHA mRNA expression in 12 BPH tissues and 20 PC tissues were detected by quantitative real-time PCR. **d** LDHA expression in Singh prostate grouped by normal prostate gland (1, *n* = 50) and prostate cancer (2, *n* = 52) derived from Oncomine database. **e** LDHA expression in Tomlins prostate grouped by no value (0, *n* = 27), BPH (1, *n* = 11), prostatic intraepithelial neoplasia (2, *n* = 13), and prostate cancer (3, *n* = 49) derived from Oncomine database. **g** Expression level of LDHA in four PC cell lines, tubulin amounts were measured as a control

To identify the expression pattern of LDHA in PC, a tissue microarray (TMA) containing 64 cases of PC specimens and 11 cases of BPH samples was analyzed using immunohistochemical staining. Higher LDHA expression was observed in 75 % (48/64) of PC cases, while 18.2 % (2/11) in BPH cases, the difference was significant (Fig. 1a, b). In addition, 20 cases of PC tissues and 12 cases of BPH tissues were collected to detect messenger RNA (mRNA) expression of LDHA using real-time quantitative PCR. We observed that LDHA mRNA expression was significantly elevated in PC tissues in relative to BPH samples (Fig. 1c). And, data from Oncomine database further confirmed this phenomenon. As shown in Fig. 1d, e, although without significant alternations in prostatic intraepithelial neoplasia, overexpressed LDHA was more commonly observed in prostate cancer. Furthermore, the expression level of LDHA in four PC cell lines was analyzed by Western blotting (Fig. 1f). To further analyze the possible functions of LDHA in PC, we selected the cell line with higher LDHA expression, PC-3 and DU145 cells, for further investigation.

### Silencing of LDHA by siRNAs inhibits cell proliferation, migration, and invasion and increases cell apoptosis in vitro

**Fig. 2:**
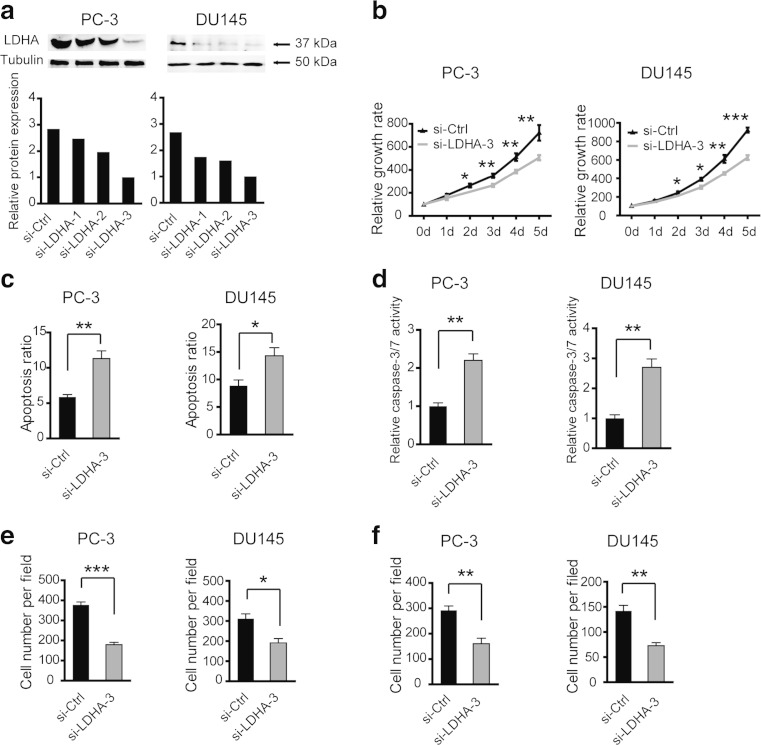
Silencing of LDHA by siRNA inhibits cell proliferation, migration, and invasion and increases cell apoptosis in vitro. **a** The expression level of LDHA was detected by Western blotting in PC-3 and DU145 cells after three different siRNA treatment. **b** Cell viability of PC-3 and DU145 cells was measured by CCK8 after LDHA knockdown. Increased cell apoptosis ratio (**c**) and caspase-3/7 activity (**d**) were observed after LDHA knockdown. Cell migration (**e**) and invasion abilities (**f**) of PC-3 and DU145 cells were decreased after LDHA was silenced. si-Ctrl versus si-LDHA-3; *P < 0.05; **P < 0.01; ***P < 0.001

The level of endogenous LDHA expression in PC-3 and DU145 cells after targeted siRNA treatment was examined by Western blotting (Fig. 2a). Given that LDHA expression was significantly decreased by siRNA-3 oligos treatment, we chose the siRNA-3 oligos for the next investigation. The CCK8 analysis showed that LDHA knockdown cells exhibited significantly reduced cell viability compared with the negative control cells (Fig. 2b). The apoptosis assay showed that cell apoptosis ratio was significantly increased after LDHA was knocked down (Fig. 2c). And, consistent with this, the caspase-3/7 activity was increased by LDHA siRNA treatment (Fig. 2d). Next, to detect whether LDHA has an implication for cancer progression, cell migration and invasion assays were performed. Migration assay showed that migrated cells in siRNA-treated group was dramatically decreased in relative to the negative control group (Fig. 2e). In the invasion assay, the invaded cells were also decreased significantly after LDHA knockdown (Fig. 2f). Collectively, these data above demonstrated that LDHA favors tumor growth and metastasis in PC.

### Inhibition of LDHA by FX11 suppresses cell proliferation, migration, and invasion and increases cell apoptosis in vitro

**Fig. 3:**
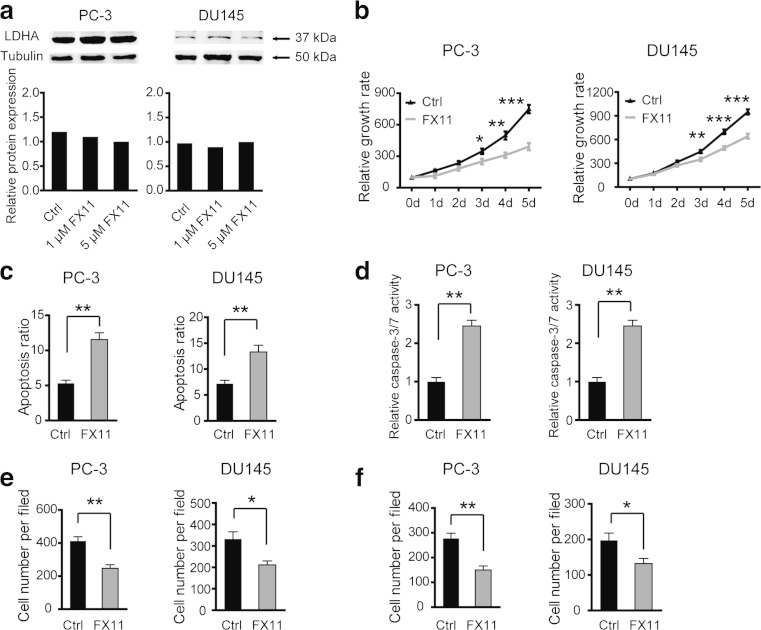
Inhibition of LDHA by FX11 suppresses cell proliferation, migration, and invasion and increases cell apoptosis in vitro. **a** The protein level of LDHA in PC-3 cells after FX11 treatment. **b** Cell viability of PC-3 and DU145 cells was measured by CCK8 after FX11 treatment. Increased cell apoptosis ratio (**c**) and caspase-3/7 activity (**d**) were observed after FX11 treatment. Cell migration (**e**) and invasion abilities (**f**) of PC-3 and DU145 cells were decreased after FX11 treatment. Ctrl versus FX11; *P < 0.05; **P < 0.01; ***P < 0.001

A small-molecule inhibitor, FX11 [3-dihydroxy-6-methyl-7-(phenylmethyl)-4-propylnaphthalene-1-carboxylic acid]), is a competitive inhibitor of LDHA. As tumor progression was inhibited by siRNA-mediated LDHA reduction, here, we evaluated the effect of FX11 in PC-3 cells. As shown in Fig. 3a, LDHA expression was not affected by FX11 treatment, indicating that FX11 mainly affect the activities of LDHA. Consistent with the results in siRNA-mediated reduction of LDHA, attenuation of LDHA activities by FX11 also resulted in decreased cell viability (Fig. 3b), increased cell apoptosis (Fig. 3c), increased caspase-3/7 activity (Fig. 3d), inhibition of cell migration (Fig. 3e), and invasion (Fig. 3f). Taken together, these results indicate that decreased LDHA enzyme activity is also involved in the antitumor effect of FX11.

### Inhibition of LDHA by siRNA or FX11 reprograms glucose metabolism

**Fig. 4:**
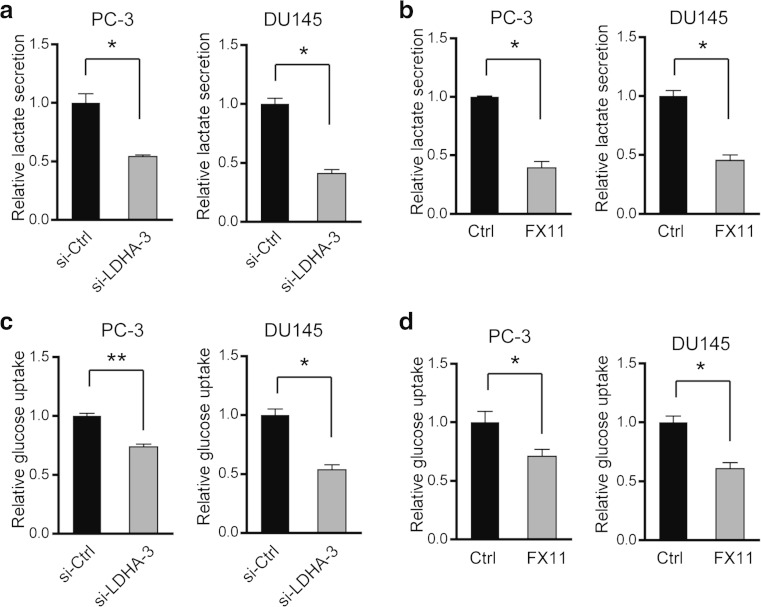
Inhibition of LDHA by siRNA or FX11 reprograms glucose metabolism. Relative lactate secretion after LDHA was knockdown (**a**) or FX11 treatment (**b**). Relative glucose consumption after LDHA was knockdown (**c**) or FX11 treatment (**d**). *P < 0.05; **P < 0.01; *P* values were calculated by the Student’s *t* test

Since LDHA executes the final step of aerobic glycolysis, we postulated that LDHA-mediated functions in PC may associate with glucose metabolism. Expectedly, the lactate level in the culture media was pronounced decreased after siRNA-mediated LDHA knockdown (Fig. 4a) or FX11 treatment (Fig. 4b). Besides, PC-3 cells also showed decreased glucose consumption after whether siRNA-mediated LDHA knockdown (Fig. 4c) or FX11 treatment (Fig. 4d). This data indicate that enhanced glycolysis may account for the tumor-suppressive effect of LDHA in PC.

### Inhibition of LDHA by siRNA or FX11 downregulates proteases involved in extracellular matrix degradation and tumor metastasis

**Fig. 5:**
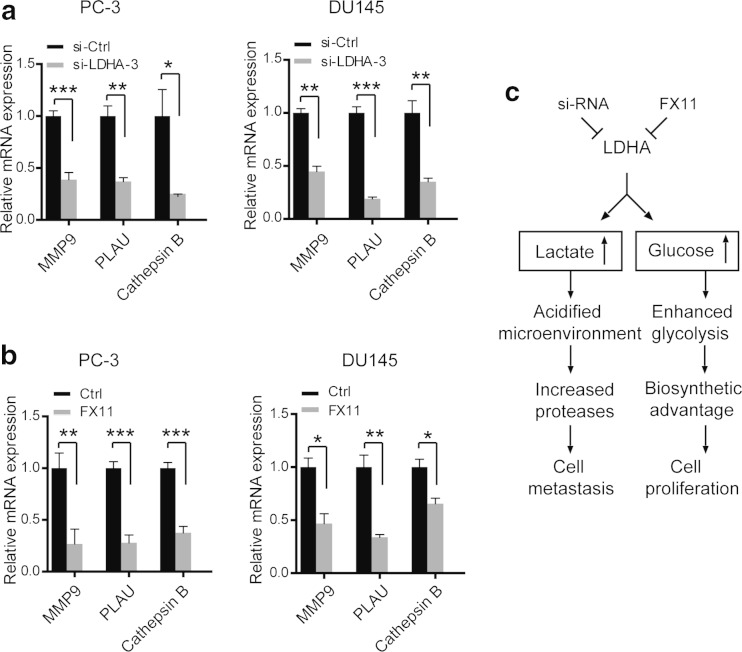
Inhibition of LDHA by siRNA or FX11 downregulates proteases involved in extracellular matrix degradation and tumor metastasis. Relative expression of MMP-9, PLAU, and cathepsin B after LDHA was knockdown (**a**) or FX11 treatment (**b**). **c** Schematic illustration of the proposed metabolic consequences induced by LDHA in PC

A critical consequence of altered lactate production and secretion is the acidification of tumor microenvironment, which favors the activation of a series of proteases, including MMP-9, urokinase type plasminogen activator (PLAU), and cathepsin B. And, this activation ultimately induces extracellular matrix degradation and facilitates tumor cells to metastasis. In our study, expression level of MMP-9, PLAU, and cathepsin B was evaluated after siRNA or FX11 treatment. Indeed, MMP-9, PLAU, and cathepsin B expression were remarkably decreased after LDHA knockdown (Fig. 5a) or FX11 treatment (Fig. 5b). In conclusion, these results support that enhanced acidified microenvironment mediated by LDHA promotes tumor cell metastasis, while increased utilization of glucose facilitates tumor cell proliferation (Fig. 5c).

## Discussion

In the present study, we observed overexpressed LDHA in PC tissues compared with BPH tissues. By using two PC cell lines, PC-3 and DU145, we asked whether the elevated LDHA was essential for tumor progression and whether LDHA altered tumor microenvironment that favors tumor growth or metastasis. To this end, we utilized siRNA to suppress the expression of LDHA and FX11 to inhibit LDHA enzyme activity. Our findings demonstrated that enhanced glycolysis and acidified microenvironment induced by LDHA had a drastic implication on tumor physiology.

Given that the accelerated glucose metabolism distinguishes cancer cells from their normal counterparts, it is reasonable to speculate that certain glycolytic enzymes are suitable to target for cancer therapy [12]. Many studies in tumors aimed at LDHA confirm this point view [6, 13, 14]. Consistent with previous reports, our results showed that knockdown of LDHA or limiting the LDHA activity of PC cells is sufficient to inhibit cell growth and metastasis [6, 9]. It is important to emphasize here that although tumor biology was altered by FX11 treatment, the LDHA enzyme activity was not measured in our study. Whether alternations induced by FX11 were due to decreased LDHA enzyme activity requires further confirmation.

It was known that although Warburg effect produces reduced ATP compared with oxidative phosphorylation (OXPHOS), it provides a constant supply of metabolites that are essential for rapid macromolecule biosynthesis and necessary for cell growth and division [15, 16]. Our results showed that inhibition of LDHA significantly compromised cell viability of PC-3 and DU145 cells and accompanied by a decrease in glucose consumption and lactate production. These results, as a proof of principle, suggest a driver role of LDHA in glycolytic activity in PC.

A critical consequence of Warburg effect is increased lactate production by tumor cells. In cancer cells, lactate was exported by monocarboxylate transporters resulting in the acidification of microenvironment, whereas this alternations leads to cell death in normal cells [17]. In our study, LDHA deficiency led to a reduction in lactate production accompanied by decreased cellular migration and invasion. Consistent with this theory, our results confirmed that the expression of proteases involved in extracellular matrix degradation including MMP9, PLAU, and cathepsin B was reduced by inhibition of LDHA. However, mechanism of this type of alternation in proteases expression induced by LDHA inhibition remains further demonstration.

In conclusion, our results from both clinical specimens and in vitro cell experiments demonstrated that LDHA expression is upregulated in PC, and it induces a favorable tumor microenvironment for tumor progression. We proposed that inhibition of LDHA may represent a novel therapeutic strategy for controlling metastasis as well as tumor growth in prostate cancer.
